# Advancements in Using AI for Dietary Assessment Based on Food Images: Scoping Review

**DOI:** 10.2196/51432

**Published:** 2024-11-15

**Authors:** Phawinpon Chotwanvirat, Aree Prachansuwan, Pimnapanut Sridonpai, Wantanee Kriengsinyos

**Affiliations:** 1 Theptarin Diabetes, Thyroid, and Endocrine Center Vimut-Theptarin Hospital Bangkok Thailand; 2 Diabetes and Metabolic Care Center Taksin Hospital Medical Service Department, Bangkok Metropolitan Administration Bangkok Thailand; 3 Human Nutrition Unit, Food and Nutrition Academic and Research Cluster, Institute of Nutrition Mahidol University Nakhon Pathom Thailand

**Keywords:** image-assisted dietary assessment, artificial intelligence, dietary assessment, mobile phone, food intake, image recognition, portion size

## Abstract

**Background:**

To accurately capture an individual’s food intake, dietitians are often required to ask clients about their food frequencies and portions, and they have to rely on the client’s memory, which can be burdensome. While taking food photos alongside food records can alleviate user burden and reduce errors in self-reporting, this method still requires trained staff to translate food photos into dietary intake data. Image-assisted dietary assessment (IADA) is an innovative approach that uses computer algorithms to mimic human performance in estimating dietary information from food images. This field has seen continuous improvement through advancements in computer science, particularly in artificial intelligence (AI). However, the technical nature of this field can make it challenging for those without a technical background to understand it completely.

**Objective:**

This review aims to fill the gap by providing a current overview of AI’s integration into dietary assessment using food images. The content is organized chronologically and presented in an accessible manner for those unfamiliar with AI terminology. In addition, we discuss the systems’ strengths and weaknesses and propose enhancements to improve IADA’s accuracy and adoption in the nutrition community.

**Methods:**

This scoping review used PubMed and Google Scholar databases to identify relevant studies. The review focused on computational techniques used in IADA, specifically AI models, devices, and sensors, or digital methods for food recognition and food volume estimation published between 2008 and 2021.

**Results:**

A total of 522 articles were initially identified. On the basis of a rigorous selection process, 84 (16.1%) articles were ultimately included in this review. The selected articles reveal that early systems, developed before 2015, relied on handcrafted machine learning algorithms to manage traditional sequential processes, such as segmentation, food identification, portion estimation, and nutrient calculations. Since 2015, these handcrafted algorithms have been largely replaced by deep learning algorithms for handling the same tasks. More recently, the traditional sequential process has been superseded by advanced algorithms, including multitask convolutional neural networks and generative adversarial networks. Most of the systems were validated for macronutrient and energy estimation, while only a few were capable of estimating micronutrients, such as sodium. Notably, significant advancements have been made in the field of IADA, with efforts focused on replicating humanlike performance.

**Conclusions:**

This review highlights the progress made by IADA, particularly in the areas of food identification and portion estimation. Advancements in AI techniques have shown great potential to improve the accuracy and efficiency of this field. However, it is crucial to involve dietitians and nutritionists in the development of these systems to ensure they meet the requirements and trust of professionals in the field.

## Introduction

### Background

Dietary assessment is a technique for determining an individual’s intake, eating patterns, and food quality choices, as well as the nutritional values of consumed food. However, this technique’s procedures are costly, laborious, and time-consuming and rely on specially trained personnel (such as dietitians and nutritionists) to produce reliable results. Consequently, a strong need exists for novel methods having improved measurement capabilities that are accurate, convenient, less burdensome, and cost-effective [1]. Instead of relying solely on client self-report, taking food photos before eating has been incorporated into traditional methods, such as a 3-day food record with food images, to reduce missing food records, incorrect food identification, and errors in portion size estimation. However, this technique still requires well-trained staff to translate food image information into reliable nutritional values and does not solve labor-intensive and time-consuming issues.

The application of computer algorithms to translate food images into representative nutritional values has gained interest in both the nutrition and computer science communities. This combination has resulted in a new field called image-assisted dietary assessment (IADA), and various systems have been developed to address these limitations, ranging from simple estimation equations in early systems to more complex artificial intelligence (AI) models in recent years. By applying IADA alongside the increasing use of smartphones and devices with built-in digital cameras, real-time analysis of dietary intake data from food images has become possible with accurate results, reduced labor, and greater convenience, thus gaining attention among nutrition professionals. However, the technical nature of this field can make it difficult to understand for those without a background in computer science or engineering, leading to the low involvement of nutrition professionals in its development. This gap is the rationale for us to conduct this review.

### Objectives

The objective of this review is to bridge that knowledge gap by providing an up-to-date overview of the gradual enhancement of AI integration in dietary assessment based on food images. The information is presented in chronological order and in a manner that is understandable and accessible to those who may not be familiar with the technical jargon and complexity of AI terminologies. In addition, the advantages and limitations of these systems are discussed. Finally, we proposed auxiliary systems to enhance the accuracy of IADA and its potential adoption within the nutrition community.

## Methods

### Overview

To conduct this scoping review, we followed the methodology suggested by Arksey and O’Malley [2] and adhered to the PRISMA-ScR (Preferred Reporting Items for Systematic Reviews and Meta-Analyses Extension for Scoping Reviews) guidelines [3].

### Search Strategy

We searched 2 web-based databases, PubMed and Google Scholar, between February 2023 and March 2023, using the following terms: ((“food image”[Title/Abstract]) AND (classification[Title/Abstract] OR recognition[Title/Abstract] OR (“computer vision”[Title/Abstract]))) and “artificial intelligence,” “dietary assessment,” “computer vision,” “food image” recognition, “portion size,” segmentation, and classification, respectively.

### Eligibility Criteria

This review included studies that focused on AI techniques used for IADA, specifically AI models, systems, or digital methods for food recognition and food volume estimation. For mobile apps or systems, we considered only articles that explain algorithms beyond mobile apps, prototype testing, or conducting clinical research. Studies that used noncomputational techniques, such as using food images as a tool for training human portion estimation, are excluded. Eligible articles were published in peer-reviewed journals or conference papers and written in English.

### Selection Process

We used Zotero (Corporation for Digital Scholarship) reference management software to collect search results using the add multiple results function. All automatic data retrieval functions were disabled to prevent data retrieval from exceeding Google Scholar’s traffic limitation. Zotero’s built-in duplicate merger was used to identify duplicated records, and unduplicated records were exported to Excel online (Microsoft Corp). In Excel, all authors independently screened article types, titles, and abstracts. The screening process removed all nonrelated titles or abstracts, review and editorial articles, non-English articles, or conference abstracts without full text. For thesis articles, the corresponding published articles were identified using keywords from the title, first author, or corresponding author whenever possible. Each article required 2 independent reviewers’ approval. In cases of conflict, a full-text review was necessary to resolve disagreements. After the initial screening process, the full texts of articles were obtained to assess eligibility. All full-text articles, whether they were excluded or not, and review articles were thoroughly read to identify interesting or related articles. These were classified as articles from other sources.

### Data Extraction

A data extraction table was constructed, including the system name, classification algorithm, portion size estimation algorithm, accuracy of classification or portion estimated results, and the system’s noticeable advantages and drawbacks. Data were extracted from full texts.

## Results

### Literature Findings

**Figure 1:**
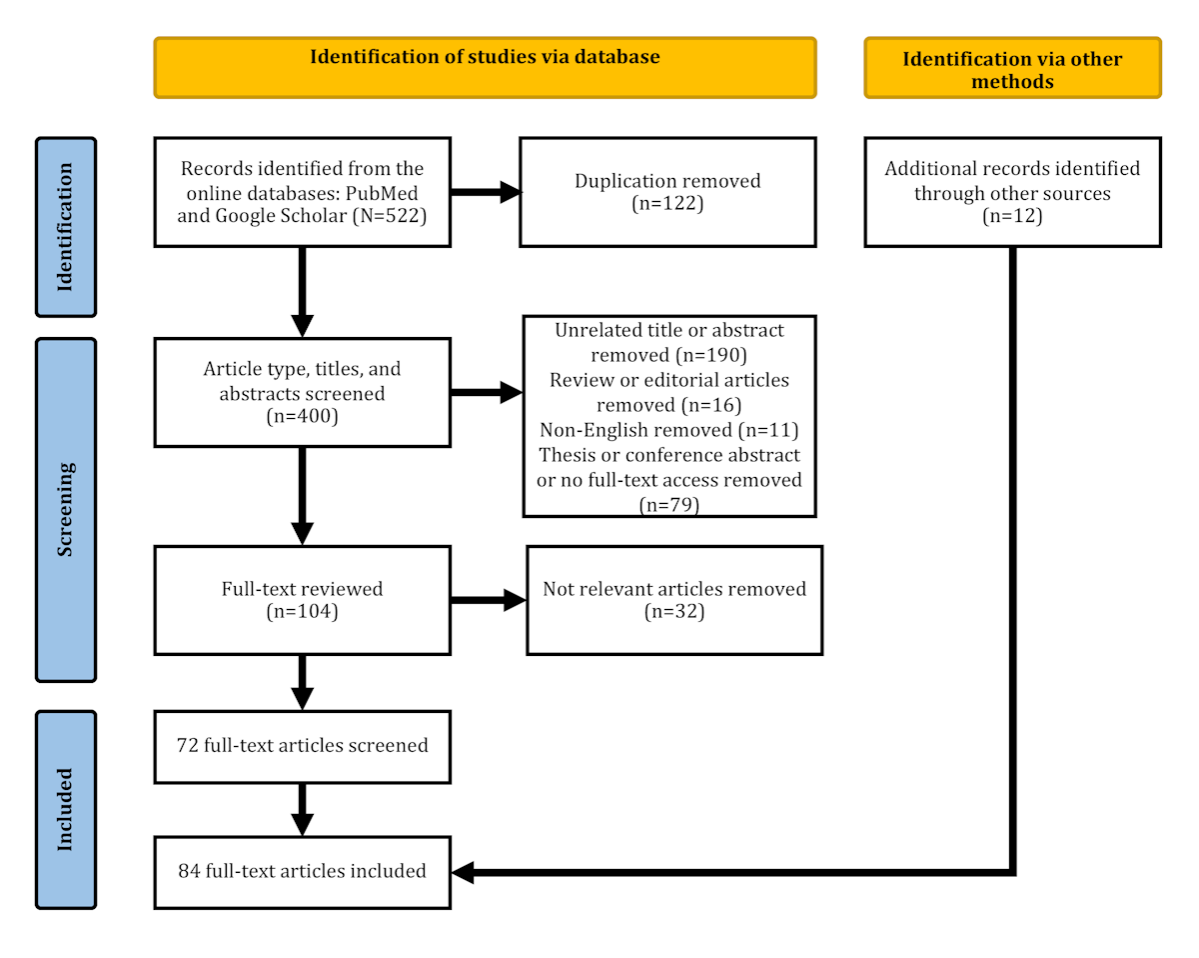
PRISMA-ScR (Preferred Reporting Items for Systematic Reviews and Meta-Analyses Extension for Scoping Reviews) flowchart of the structured literature search, screening, and selection methodology.

We retrieved 44 (8.4%) items from PubMed, while Google Scholar provided 478 (91.6%) results from the search terms, giving a total of 522 items retrieved. In total, 122 (23.4%) duplicate items were removed using Zotero’s built-in duplicate merger. The remaining 400 (76.6%) deduplicated items were screened based on their titles and abstracts, resulting in 104 (19.9%) records for full-text review. After the full-text review process, 72 (13.8%) articles were included in this study. In addition, we manually identified and included 12 (2.3%) additional articles from other sources. An overview of the literature identification method and results is shown in Figure 1, and the PRISMA-ScR checklist is available in Multimedia Appendix 1.

### Traditional Dietary Assessment Methods

When measuring individual food intake, dietary assessment methods are typically divided into 2 sequential processes: methods to obtain dietary intake and methods to estimate the nutritional values of food. Principally, obtaining an individual’s intake can be done by recording all consumed foods, beverages, herbs, or supplements with their portion sizes on a day-to-day basis or within a specific time frame (eg, a week) based on variation in the nutrients of interest. These methods were developed early on and can be performed manually. Due to their simplicity, some methods are frequently used in nutrition professionals’ practices.

The 24-hour dietary recall (24HR) method is the simplest way to measure dietary intake, but accurately obtaining dietary intake information can be very challenging. The participant or their caregiver are asked by a trained interviewer to recall the participant’s food intake within the last 24 hours. This method relies heavily on the client’s memory and estimation of food portion size [4]. Unintentional misreporting of food intake is common, as clients often forget some foods. Underreporting of portion size is common because clients are not familiar with estimating food portion sizes [5,6]. In participants who are overweight or obese, intentional underreporting is also common [7]. Although this method is the simplest for determining dietary intake, it takes approximately 1 hour to complete each interview. Moreover, a single 24HR result does not satisfactorily define an individual’s usual intake due to day-to-day variations in eating habits.

Estimated food records (EFRs) are more reliable but time-consuming. Clients are asked to record all food and beverage intake during eating times for a specified period. Details of food are needed along with the portion sizes estimated by the client and rounded to household units (eg, half cup of soymilk with ground sesame and 4 tablespoons of kidney beans without syrup). To improve accuracy, training in estimating portion size using standard food models is required. The EFR places a burden on the clients, as they need to record all eating times. Moreover, some clients temporarily change their intake habits during recording to minimize this burden, while others may intentionally not report certain foods to cover up certain eating habits. Food portion size estimation errors are sometimes found, but taking food photographs before and after eating can lower these errors [8-12].

A standardized weighing scale can be used to avoid errors caused by human estimation of portion sizes. This technique is known as weighed food records and is considered the gold standard for determining personal intake. However, it is impractical to weigh all eaten food in the long term because it becomes a burden for the client to measure the weight of food eaten throughout the day [4]. This technique also only eliminates portion size estimation errors, while other issues with EFRs may still persist.

After retrieving dietary intake information from sources, such as 24HR, EFR, or weighed food records, the next step is to estimate the representative nutritional value of the food using a food composition table. If the recorded foods match the food items and their description in an available food composition table, the nutritional values can be obtained by multiplying the consumed food weight directly. However, if the food items are not found, the food needs to be analyzed and broken down into its components. The nutritional values of each component can then be obtained from the food composition table (or its nutrition label) and multiplied by the actual weight of each consumed component. When the portion size is recorded instead of its actual weight, the estimated weight can be obtained using standardized portion sizes from the food composition table. Nutrient analysis software can easily accomplish this task.

### IADA Methods

#### Overview

Digital devices are often used for dietary assessment. The first well-documented attempt to develop such a digital device was called Wellnavi by Wang et al [8]. Although the device yielded accurate results, its usability was limited by the technologies of the time, including short battery life, poor image quality, a bulky body, and a less sensitive touch screen [10].

Several attempts have been made to use generic devices, such as Palm (Palm Inc) PDAs [13], compact digital cameras [14], and smartphones [15], instead of inventing a specific food recording device. In using these devices, users reported a decrease in the burden of completing food recording when compared with traditional methods [16,17]. However, these devices still rely heavily on dietitians or nutritionists to analyze the nutritional values of food items.

Recent advancements in mobile phone technologies, including high-performance processors and high-quality digital cameras, have created the opportunity to invent a food image analysis system on smartphones. While the exact origins of applying AI for IADA research are uncertain, one well-documented attempt to develop a simple system on smartphones was that of DiaWear [18]. The system implemented an artificial neural network, which is a subset of deep learning, a recently advanced technique in the field of AI. Despite achieving an accuracy rate above 75%, which was considered incredible at that time, the system’s usefulness was limited because it could identify only 4 types of foods—hamburgers, fries, chicken nuggets, and apple pie. In addition, the system could not determine the portion size of the taken food image; thus, it gave a nutritional value based on a constant portion size directly.

In this paper, the architecture of IADA is divided into multistage architectures, which were prevalent in the early stages of IADA development, and end-to-end architecture, which has emerged more recently with advancements in AI techniques and food image datasets. The multistage architectures, as implied by their name, include 4 individual processes: segmentation, food identification, portion estimation, and nutrient calculations using a food composition table. This sequential process is consistent across all early-stage IADA systems [19-23]. These subprocesses are trained independently because they require specific input variables, and optimization can only be done for each step individually, not for the entire process. By contrast, the end-to-end approach, which replaces a multistep pipeline with a single model, can be fine-tuned as a whole process, making it more advanced and increasingly the focus of researchers today.

**Figure 2:**
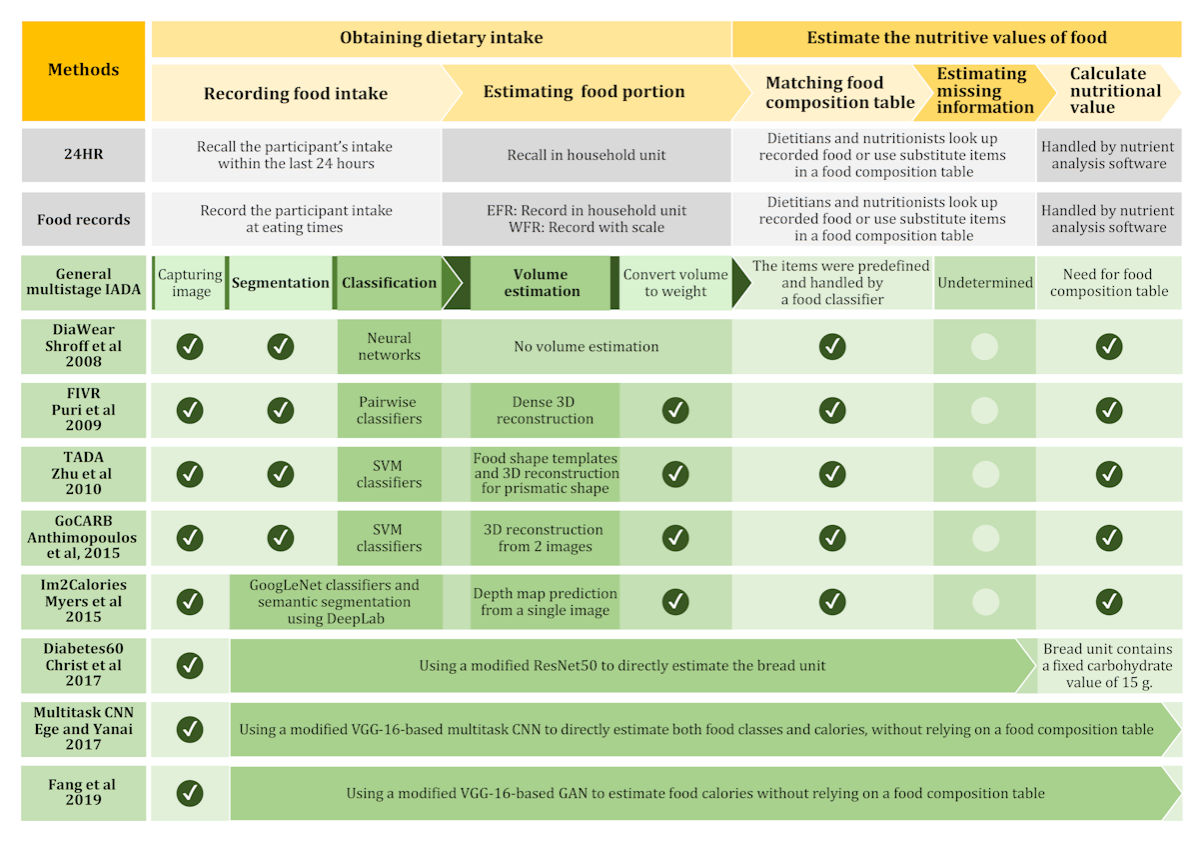
Comparison of traditional dietary assessment processes and the image-assisted dietary assessment (IADA) substitution processes for the same tasks, including systems that integrate multistage architecture into a single model using deep learning. Systems referenced include DiaWear from Shroff et al [18], GoCARB from Anthimopoulos et al [23], FIVR from Puri et al [26], Im2Calories from Myers et al [27], Diabetes60 from Christ et al [28], Multitask CNN from Ege and Yanai [29], Fang et al [30], and technologies-assisted dietary assessment (TADA) from Zhu et al [24, 25,31]. 24HR: 24-hour dietary recall; CNN: convolutional neural network; EFR: estimated food record; GAN: generative adversarial network; ResNet50: residual network; SVM: support vector machine; VCG: visual geometry group; WFR: weighed food record.

Nowadays, multistage architectures are becoming obsolete and are often referred to as traditional IADA. They played a significant role in the IADA timeline before the emergence of the end-to-end approach. Therefore, we delve into the multistage architectures, particularly focusing on food identification and portion estimation algorithms in their subsections, and provide details about the end-to-end approach in the Going Beyond the Traditional Approach With Deep Learning section. For better comparison, Figure 2 illustrates traditional dietary assessment methods and the substitution processes of IADA, along with some notable systems that indicate combining certain processes of the multistage architecture into a single model through deep learning [18,23-31].

#### Food Identification System

Image recognition systems are one of the milestones in the computer vision field. The goal is to detect and locate an interesting object in an image. Several researchers have applied this technique to food identification tasks that formerly relied on humans only. The early stages in the development of food identification systems were from 2009 to 2015. Most of the existing systems were powered by machine learning algorithms that required human-designed input information, or technical terms called features. Hence, all machine learning-based algorithms are classified as handcrafted algorithms.

The era of handcrafted algorithms began in 2009 with the release of the Pittsburgh Fast-Food Image Dataset [19], marking a significant historical landmark in promoting research into food identification algorithms. This dataset consisted of 4545 fast-food images, including 606 stereo image pairs of 101 different food items. In addition, researchers provided baseline detection accuracy results of 11% and 24% using only the image color histogram together with the support vector machines (SVMs)-based classifier and the bag-of-scale-invariant feature transform classifier, respectively. Although these classifiers were commonly used during that time, the results were not considered sufficient and demonstrated much room for improvement. Since then, various techniques have been proposed to improve the accuracy of food classification from images. In later studies, the same team used pairwise statistics to detect ingredient relations in food images, achieving an accuracy range of 19% to 28% on the Pittsburgh Fast-Food Image Dataset [20]. Taichi and Keiji [21], from the University of Electro-Communications (UEC) team, used multiple kernel learning, which integrates different image features such as color, texture, and scale-invariant feature transform. This method achieved 61% accuracy on a new dataset of 50 food images and 37.5% accuracy on real-world images captured using a mobile phone [21]. In 2011, Bosch et al [22] from the Technology Assisted Dietary Assessment (TADA) team achieved an accuracy of 86.1% for 39 food classes by using an SVM classifier. This approach incorporated 6 features derived from color and texture [22]. These results suggest that including a larger number of features in the algorithms could potentially improve detection accuracy.

After active research, the accuracy of handcrafted algorithms reached a saturation point for improvement during the 2014 period. The optimized bag-of-features model was applied to food image recognition by Anthimopoulos et al [23]. It achieved an accuracy level of up to 77.8% for 11 classes of food on a food image dataset containing nearly 5000 images for the type 1 diabetes project called GoCARB. Pouladzadeh et al [32] achieved a 90.41% accuracy for 15 food classes using an SVM classifier with 4 image features: color, texture, size, and shape. Kawano and Yanai [33] (UEC) attained a 50.1% accuracy for a new dataset comprising 256 food classes, using a one-vs-rest classifier with a Fisher vector and a derived feature from a color histogram named RootHoG [33]. While handcrafted algorithms yielded high-accuracy results for their specific test datasets with fewer food classes, they struggled to effectively handle larger class sets and real-world images. This difficulty arose due to factors, such as challenging lighting conditions, image noise, distorted food shapes, variations in food colors, and the presence of multiple items within the same image. Handcrafted algorithms may reach a limitation in their ability to improve further.

In contrast, the novel approach called deep learning, which can automatically extract features from input data, appears to be more suitable for complex tasks such as food identification. The convolutional neural network (CNN), considered to be one of the approaches in deep learning, was developed for handling image analysis in 1998 [34]. CNN reads a group of squared pixels of an input image, referred to as a receptive field, and then applies a mathematical function to the read data. The operation is performed repeatedly from the top-left corner until reaching the bottom-right corner of an input image. This operation is done in a similar manner to matrix multiplication or dot product in linear algebra. CNN and deep learning were applied to the food identification task in 2014 by the UEC team [35]. This system achieved an accuracy of 72.3% on a dataset containing 100 classes of real-world Japanese food images, named UEC FOOD-100, surpassing their previous handcrafted system in 2012, which achieved 55.8% on the same dataset [36]. This marked the beginning of the era of applying deep learning techniques for food identification. Later that year, the UEC team also released an international food image dataset called UEC FOOD-256 that contained 256 food classes to facilitate further research [37]. Simultaneously, the FOOD-101 dataset was made available, comprising nearly 101,000 images of 101 different food items [38]. They also presented baseline classification results from the random forest–based algorithm, one of the handcrafted algorithms, and compared it with CNN. They found that CNN achieved an accuracy of 56.4%, while random forest–based algorithm achieved 50.76% accuracy in this dataset. These food image datasets have become the favored benchmark for subsequent food identification systems.

Another important technique is transfer learning, which is well-known for training many deep learning algorithms, including CNNs. It involves 2 stages: pretraining and fine-tuning. Initially, the model is trained with a large and diverse image dataset, and then it is further trained with a smaller, more specific dataset to enhance detection accuracy. This approach is similar to how humans are educated, where broad knowledge is learned in school followed by deeper knowledge in university. The UEC team applied this training approach to the food identification task in 2015 and successfully achieved an accuracy of 78.77% on the UEC FOOD-100 dataset [39]. It has been reported that pretraining on large-scale datasets for both food and nonfood images could improve the classification system’s accuracy beyond 80% [40-45], which is considered to surpass all handcrafted algorithms and be sufficient for real-world applications.

Currently, numerous state-of-the-art object detectors or classifier models, including the pretrain and fine-tune training paradigm, have been developed and are available, such as AlexNet (AlexNet is an object detection model that won the ImageNet Challenge in 2012; it is named after its inventors, Alex Krizhevsky) [46], region-based CNN (R-CNN; an object detection model that significantly improved object detection performance by combining region proposals with CNNs) [47], residual network (ResNet; a deep learning model that won the ImageNet Challenge in 2015, known for its innovative use of residual learning to train very deep networks) [48], You Only Look Once (YOLO; it is an object detection model that introduced a novel approach by framing object detection as a single regression problem, predicting bounding boxes and class probabilities directly from full images in one step evaluation) [49], Visual Geometry Group (VGG) [50], and Inception (this is an object detection model that won the ImageNet Challenge in 2014, recognized for its use of a novel architecture that efficiently leverages computing resources inside the network) [51]. These object detectors have been designed to automatically extract features from input images and learn distinct characteristics of each class during the training process. Deep learning-based object detection models have shown great promise in image recognition tasks, especially in complex tasks such as food identification. These models and their derivatives are commonly found in many of the food identification systems developed later. The use of these state-of-the-art models presents an exciting opportunity for nutrition researchers who may not have a background in computer engineering or data science. They can now create high-performance food identification systems for specific tasks by curating a food image dataset and training the model accordingly. With the various algorithms available, it is crucial to carefully consider their unique characteristics to select the most suitable one for a given application. The notable food identification systems are listed in Table 1.

**Table 1:** Overview of notable food identification systems, classifier algorithms, selected features, number of classes, name of food dataset (if specified or noted as their own dataset if absent), and accuracy results^a^.

Study, year	Projects or team	Classifier	Feature	Class (dataset)	Accuracy results percentages
Shroff et al [18], 2008	DiaWear	Neural network	Color, size, shape, and texture	4	~75
Chen et al [19], 2009	PFID^b^	SVM^c^	Color BoSIFT^d^	61 (PFID)	~11 ~24
Taichi and Keiji [21], 2009	UEC^e^	MKL^f^	Color, texture, and SIFT^g^	50	61.34
Hoashi et al [52], 2010	UEC	MKL	BoF^h^, Gabor^i^, color, HOG^j^, and texture	85	62.53
Yang et al [20], 2010	PFID	SVM	Pairwise local features	61 (PFID)	78.00
Zhu et al [31], 2010	TADA^k^	SVM with Gaussian radial basis kernel	Color and texture	19	97.20
Kong and Tan [53], 2011	DietCam	Multiclass SVM	Nearest neighbor Gaussian region detector, and SIFT	61 (PFID)	84.00
Bosch et al [22], 2011	TADA	SVM	Color, entropy, Gabor, Tamura^l^, SIFT, Haar wavelet^m^, steerable^n^, and DAISY^o^	39	86.10
Matsuda et al [36], 2012	UEC	MKL-SVM	HOG, SIFT, Gabor, color, and texture	100 (UEC-Food100)	55.80
Anthimopoulos et al [23], 2014	GoCARB	SVM	HSV^p^-SIFT, optimized BoF, and color moment invariant	11	78.00
He et al [54], 2014	TADA	k-nearest neighbors	DCD^q^, SIFT, MDSIFT^r^, and SCD^s^	42	65.4
Pouladzadeh et al [32], 2014	—^t^	SVM	Color, texture, size, and shape	15	90.41
Kawano and Yanai [35], 2014	UEC	Pretrained CNN^u^	—	100 (UEC-Food100)	72.3
Yanai and Kawano [39], 2015	UEC	Deep CNN	—	100 (UEC-Food-100)	78.77
Christodoulidis et al [40], 2015	GoCARB	Patch-wise CNN	—	7	84.90
Myers et al [27], 2015	Google	GoogLeNet	—	101	79.00
Liu et al [41], 2016	—	DeepFood	—	Food-101 UEC-256	77.40 54.70
Singla et al [42], 2016	—	GoogLeNet	—	11	83.60
Hassannejad et al [43], 2016	—	InceptionV3^v^	—	101 (Food-101) 100 (UEC-Food100) 256 (UEC-Food256)	88.28 81.45 76.17
Ciocca et al [44], 2017	—	VGG^w^	—	73 (UNIMINB2016)	78.30
Mezgec and Koroušić Seljak [45], 2017	—	NutriNet (Modified AlexNet^x^)	—	73 (UNIMINB2016)	86.72
Pandey et al [55], 2017	—	Ensemble net	—	101 (Food-101)	72.10
Martinel et al [56], 2018	—	WISeR^y^	—	101 (Food-101) 100 (UEC-Food100) 256 (UEC-Food256)	88.72 79.76 86.71
Jiang et al [57], 2020	—	MSMVFA^z^	—	101 (Food-101) 172 (VireoFood-172) 208 (ChineseFoodNet)	~90.47 90.61 81.94
Lu et al [58], 2020	GoCARB	Modified InceptionV3	—	298 Generic food Subgroups Fine-grained (MADiMA^aa^)	65.80 61.50 57.10
Wu et al [59], 2021	—	Modified AlexNet	—	22 styles of Bento sets	96.30

^a^Note that convolutional neural network–based classifiers do not require the number of features to be shown as they extract features autonomously.

^b^PFID: Pittsburgh Fast-Food Image Dataset.

^c^SVM: support vector machine.

^d^BoSIFT: bag-of-scale-invariant feature transform.

^e^UEC: University of Electro-Communications.

^f^MKL: multiple kernel learning. This is a machine-learning technique that combines multiple kernels or similarity functions, to improve the performance and flexibility of kernel-based models such as support vector machines.

^g^SIFT: scale-invariant feature transform.

^h^BoF: bag-of-features.

^i^Gabor is a texture feature extraction invented by Dennis Gabor.

^j^HOG: histogram of orientated gradients—a feature descriptor based on color.

^k^TADA: Technology Assisted Dietary Assessment.

^l^Tamura is a 6-texture feature extraction invented by Hideyuki Tamura.

^m^Haar wavelet is a mathematical analysis for wavelet sequence named after Alfréd Haar.

^n^Steerable filter is an image filter introduced by Freeman and Adelson.

^o^DAISY is a local image descriptor introduced by E Tola et al [60], but they did not describe a true acronym of DAISY.

^p^HSV is the name of a red-green-blue color model based on hue, saturation, and value.

^q^DCD: dominant color descriptor.

^r^MDSIFT: multiscale dense scale-invariant feature transform.

^s^SCD: scalable color descriptor.

^t^Not available.

^u^CNN: convolutional neural network.

^v^Inception is an object detection model that won the ImageNet Challenge in 2014, recognized for its use of a novel architecture that efficiently leverages computing resources inside the network.

^w^VGG: visual geometry group—an object detection model named after a research group from the University of Oxford.

^x^AlexNet is an object detection model that won the ImageNet Large-Scale Visual Recognition Challenge (also known as the ImageNet challenge) in 2012; it is named after its inventors, Alex Krizhevsky.

^y^WISeR: wide-slice residual.

^z^MSMVFA: multi-scale multi-view feature aggregation.

^aa^MADiMA: Multimedia Assisted Dietary Management.

#### Food Portion Size Estimation System

##### Overview

**Figure 3:**
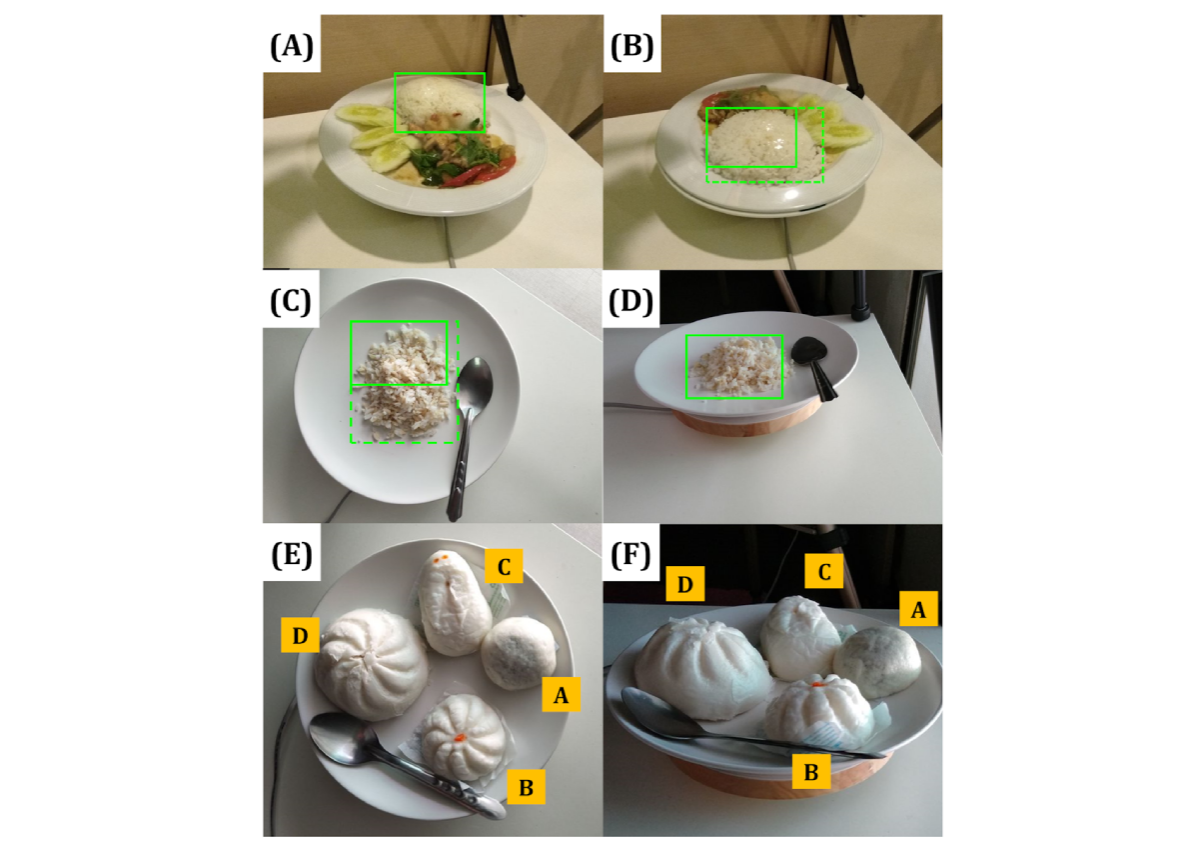
There are common image perspective distortion problems. Firstly, position distortion: the size of the white rice in (A) is smaller compared to (B) because the white rice in (B) is closer to the camera. Secondly, angle distortion: the white rice in (C) is fully visible at 90 degrees, while it appears smaller when taken from 30 degrees, as in (D). Thirdly, there is a loss of depth information in the bird’s-eye view in (E) and (F), making it difficult to compare food B and food C.

Food portion size estimation is a challenging task for researchers as it requires more accurate information on the amount of food, ingredients, or cooking methods that cannot be obtained from only a captured image without additional input, which makes it harder to create a food image dataset with portion size annotation. Furthermore, quantifying an object’s size from a single 2D image is faced with common image perspective distortion problems [61,62], as shown in Figure 3. First, the size of the object in the image can change due to the distance between the object (food) and the capturing device (smartphone or camera). The size of the white rice in Figure 3A is smaller compared with Figure 3B because the white rice in Figure 3B is closer to the camera. Second, the angle at which the photo is taken also alters the perceived object size. For example, flattened objects such as rice, that are spread out on a 23-cm (9-inch) circular plate appear in their full size in a bird’s-eye shot (90°), in Figure 3C, but they appear smaller when taken from approximately 30° from the tabletop as in Figure 3D. Thirdly, there is a loss of depth in a bird’s-eye view in Figures 3E and 3F, making it difficult to compare between food B and food C. The weights of foods A, B, C, and D are 48, 49, 62, and 149 grams, respectively. We use these images for teaching image-based portion estimation for dietetics students.

While pretrain and fine-tune training for CNNs is a silver bullet for food image identification, currently there is no equivalent solution for portion estimation. Many researchers are actively finding ways to calibrate the object size within an image to mediate such an error, and several approaches have been discussed here. Basically, portion estimation can be broadly classified, based on complexity, into four progressive categories: (1) pixel density, (2) geometric modeling, (3) 3D reconstruction, and (4) depth camera. Table 2 provides an overview of notable systems for volume estimation.

**Table 2:** A comprehensive overview of notable publications for 4 volume estimation approaches, arranged chronologically.

Approach and study, year		Projects or team	Reference object	Item	Reported error
**Pixel density approach**					
	Martin et al [13], 2009	—^a^	Physical card	N/A^b^	N/A
	Jia et al [63], 2012	University of Pittsburgh	Circular plate Circular LED light	—	<27.60 <54.10
	Pouladzadeh et al [32], 2014	—	User’s thumb	5	<10
	Okamoto and Yanai [64], 2016	UEC^c^	Wallet	3	Mean calorie error Beef rice bowl –242 (SD 55.1) Croquette –47.08 (SD 52.5) Salad 4.86 (SD 11.9)
	Akpa et al [65], 2017	—	Chopstick	15	<6.65
	Liang and Li [66], 2017	—	1-yuan coin	19 fruits	15 items <20%
	Yanai et al [67], 2019 and Ege et al [67], 2019	UEC	Rice grain size	3	<10%
**Geometric modeling approach**					
	Zhu et al [24], 2010 and Zhu et al [25], 2008	TADA^d^	Checkerboard	7	Spherical 5.65% Prismatic 28.85%
	Chae et al [69], 2011	TADA	Checkerboard	26	Cylinders 11.1% Flattop solid 11.7%
	Chen et al [70], 2013	University of Pittsburgh	Circular plate	17	3.69%
	Jia et al [71], 2014	University of Pittsburgh	Circular plate Other container	100	<30% from 85/100 of test items
	Tanno et al [72], 2018	UEC	Apple ARKit	3	Mean calorie error Beef rice bowl –67.14 (SD 18.8) Croquette–127.0 (SD 9.0) Salad –0.95 (SD 0.16)
	Yang et al [73], 2019	University of Pittsburgh	Augmented reality	15	Large objects 16.65% Small objects 47.60%
	Smith et al [74], 2022	—	Checkerboard	26	Single food items 32.4%-56.1% Multiple food items 23.7%-32.6%
**3D reconstruction approach**					
	Puri et al [26], 2009	—	3 images Checkerboard	26	2%-9.5%
	Kong and tan [75], 2012	—	3 images Checkerboard	7	Volume estimation error 20%
	Rahman et al [76], 2012	TADA	2 images Checkerboard	6	7.70%
	Chang et al [77], 2013	TADA	Using food silhouettes to reconstruct a 3D object	4	10%
	Anthimopoulos et al [78], 2015	GoCARB	2 images physical card Physical card	N/A	Volume estimation error 9.4%
	Dehais et al [79], 2017	GoCARB	2 images Physical card	45 dishes 14 meals	8.2%-9.8%
	Gao et al [80], 2018	—	SLAMe-based with Rubik cube	3	11.69%-19.20% for static measurement 16.32%-27.9% for continuous measurement
	Ando et al [81], 2019	UEC	Multiple cameras on iPhone X for depth estimation	3	Calorie estimation error Sweet and sour pork <1% Fried chicken <1% Croquette <15%
	Lu et al [58], 2020	GoCARB	2 images Physical card and gravity information	234 items from MADiMA^f^	MARE^g^ 19%, while their earlier system, GoCarb (2017), achieved 22.6% on the same task [79].
**Depth camera approach**					
	Shang et al [82], 2011	—	Specific food recording device	—	No performance report
	Chen et al [83], 2012	—	Depth camera	—	No performance report
	Fang et al [84], 2016	TADA	Camera from this study [85]	10	Depth method overestimates volume than geometric model
	Alfonsi et al [86], 2020	—	iPhone and Android devices	200	Carbohydrate estimation error <10 g
	Herzig et al [87], 2020		iPhone X	128	Relative error of weight estimation 14.0%

^a^Not available.

^b^N/A: not applicable.

^c^UEC: University of Electro-Communications.

^d^TADA: Technology Assisted Dietary Assessment.

^e^SLAM: simultaneous localization and mapping.

^f^MADiMA: Multimedia Assisted Dietary Management.

^g^MARE: mean absolute relative error.

##### Revisiting the Classic Pixel Density Approach

Pixel density is the simplest approach for providing good and effective estimation. After a food image is segmented, the number of pixels in each segmented section is determined. Mathematical equations or other transformations are then used to calculate the portion size of each section that is presented in the image.

However, this approach suffers from image distortion problems, and several approaches have been implemented to combat this drawback. The simplest method is the use of a physical reference object or fiducial marker for calibrating the size of objects in an image. When the real size of the reference object is known, the real size of an object can be determined relative to the reference object. This method was chosen for food volume estimation during its early development stage [13,88,89]. Various physical objects have been used as reference objects in the literature, including a special patterned card [13,89], a known-size circular plate [63] or bowl [90], chopsticks [65], a 1-yuan coin [66], a wallet [64], a user’s thumb [40,91], or even rice grain size [67].

##### Geometric Modeling Approach

**Figure 4:**
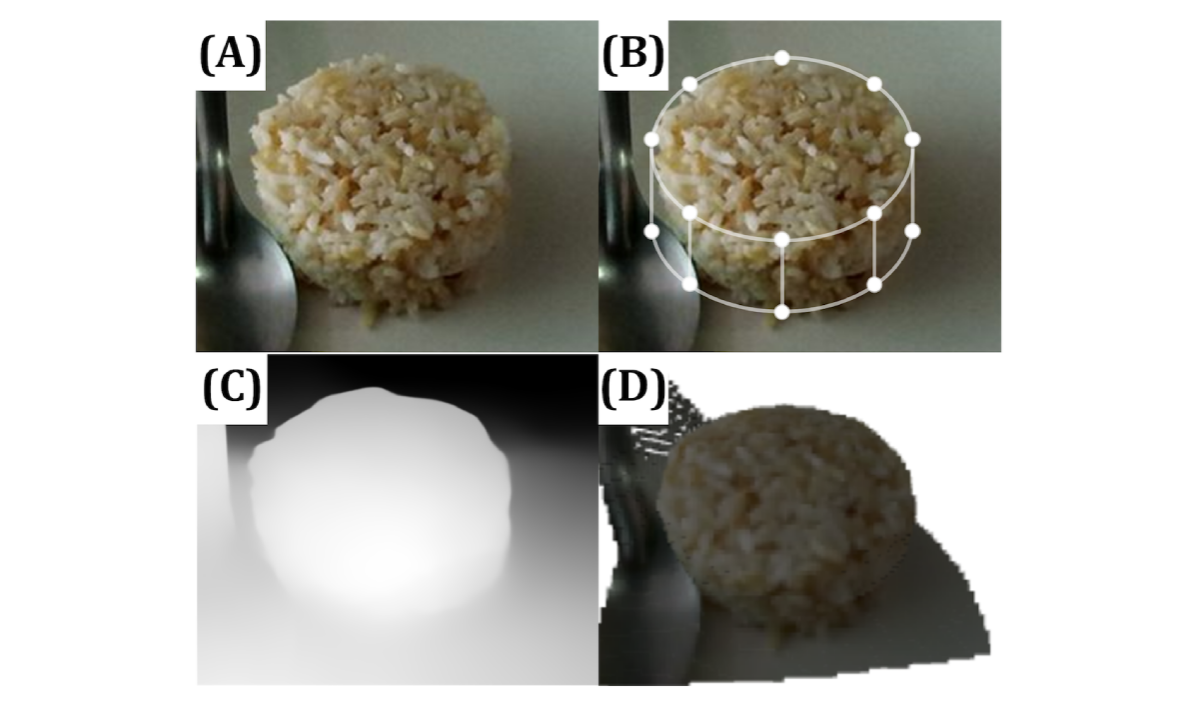
This figure demonstrates the various approaches to estimating food volume. (A) A cylindrical shape of 75 grams of brown rice taken from a 60° angle. (B) Geometric modeling with a predefined cylindrical shape, where the user needs to adjust each point manually to fit the object. (C) A predicted depth map from state-of-the-art dense prediction transformation. (D) A 3D reconstructed object using depth information from (C). These images have been adjusted in size for visual comparison purposes.

Assuming that the food has a cylindrical shape, such as compressed steamed rice (Figure 4A), its volume can be calculated using the conventional formula 2πr^2^ × h. The radius r and height h can be determined by counting the pixels in the image. While this approach is effective for geometric shapes, it is less reliable for irregular shapes that lack a specific equation. The demonstration of this approach is shown in Figure 4B, where the user selects a predefined shape and then manually fits (or registers) the geometric model with the image.

The TADA team reported the use of several predefined shapes of foods, including cylindrical, flattop solid, spherical, and prismatic models [24,25,68,69]. Prismatic models were specifically used to estimate portion sizes of irregularly shaped foods. This approach allowed a more accurate estimation of portion sizes by considering the unique characteristics of each food item. The research team at the University of Pittsburgh proposed a similar technique known as wireframe modeling. This technique involves creating a skeletal representation of an object using lines and curves to define its structure to accurately capture the shape and dimensions of food items [70,71]. However, this approach is also affected by common image distortion problems. Initially, a physical reference object was used for calibration.

**Figure 5:**
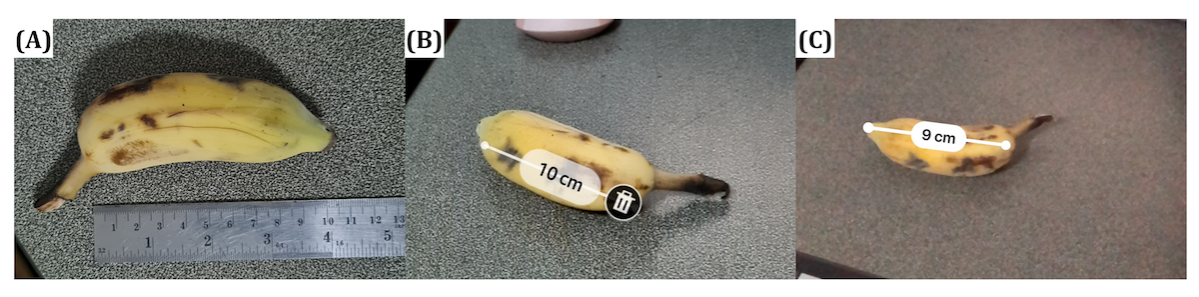
Measuring the size of the same banana can be done using different techniques, as shown in the figure. (A) A standard ruler is used as a ground truth measurement, (B) Samsung augmented reality Zone app, and (C) Apple iPhone Measure app. These apps use the gyroscope or accelerometer sensors in the mobile phone to accurately track the movement of the phone as the measurement line is drawn.

Geometric modeling shares a fundamental principle with augmented reality (AR), a technology that transforms 2D environmental images into 3D coordinates in a computer system. As AR has become more widely available on smartphones, many researchers have explored the feasibility of using AR as a calibration method instead of using physical reference objects [72,73]. AR-based object length measurement is demonstrated in Figure 5.

##### 3D Reconstruction

This technique involves using ≥2 images taken from different angles to create virtual 3D objects in 3D coordinates in a computer system. It shares the same principle as both AR and geometric modeling, where reconstructed objects are represented similarly to prismatic models in geometric modeling. Furthermore, this technique allows for the inclusion of shapes beyond traditional geometric shapes.

While several researchers have explored the use of 3D reconstruction [26,75,76], 1 notable example is the GoCARB system [78]. This system requires 2 images taken from different angles to construct a 3D model of the food, achieving an accuracy within 20 grams for carbohydrate content estimation. This level of accuracy is comparable to estimates made by dietitians when the food is completely visible on a single dish with an elliptical plate and flat base [92].

Figures 4C and 4D demonstrate a similar 3D reconstruction approach but implemented using state-of-the-art dense prediction transformation models to predict depth maps from a single image (Figure 4A), followed by the reconstruction of the 3D object using the predicted depth map.

##### Depth Camera Approach

This method operates on the same principle as geometric modeling and 3D reconstruction, but it requires a special time-of-flight (ToF) sensor (also known as a depth camera) to measure an object’s size in 3D coordinates in a computer system. Initially, the application of depth cameras in food volume estimation was limited, primarily due to their high cost and limited availability [82]. However, with the introduction of consumer-grade depth cameras, such as Kinect (Microsoft Corp), Intel RealSense, and smartphones equipped with depth sensors, their accessibility increased, leading to wider use in food volume estimation applications [81,83,84,86,87].

Nevertheless, the availability of depth sensors remains a significant challenge in implementing this system. Currently, only a limited number of mobile phone models are equipped with such sensors. In addition, some manufacturers integrate the sensor with the front camera for authentication purposes, such as Apple’s FaceID, making it impractical for capturing object photos. Moreover, certain mobile device manufacturers have omitted the ToF sensor in their recent models [93], further reducing the availability of depth sensors and posing implementation challenges for the depth camera approach.

**Figure 6:**
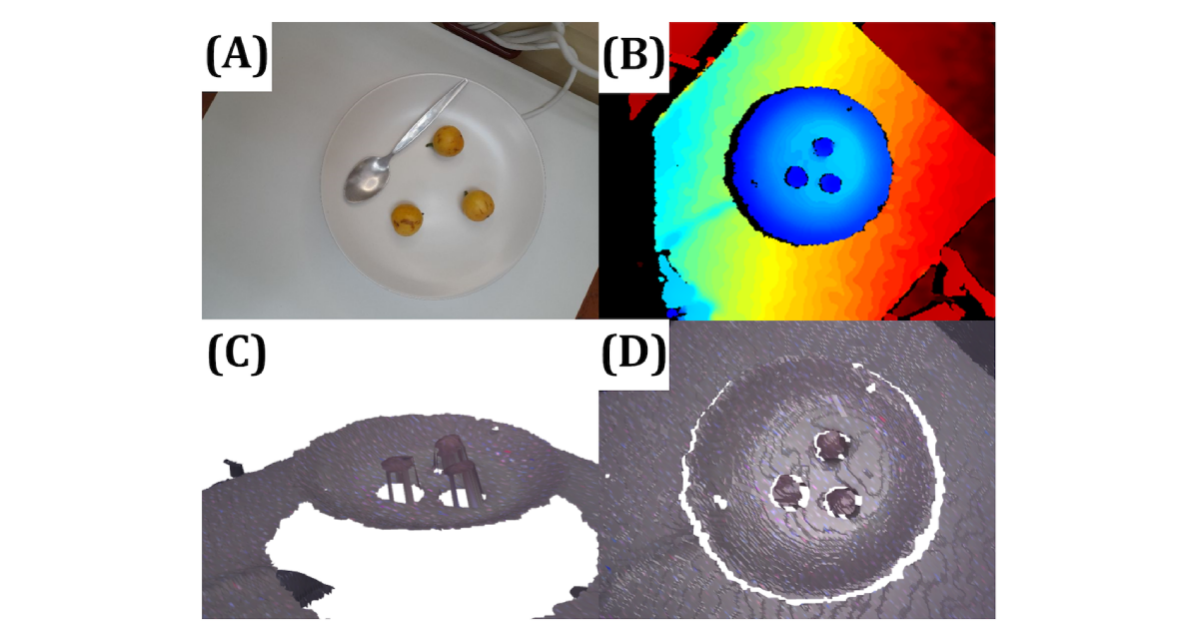
(A) A typical red-green-blue image showing 3 Burmese grapes, each weighing approximately 20 grams. (B) A red-green-blue image with depth captured by Intel RealSense d435i from a bird’s-eye view. (C) and (D) 3D reconstructed objects from the polygon file, illustrating the height of each fruit from different angles.

An example of depth information captured by the Intel Realsense d435i depth camera displayed in RGB (red-green-blue; color model based on additive color primaries) with depth (RGB with depth; RGBD) format is shown in Figure 6B. Rendered objects from a captured polygon file are demonstrated as freely rotatable 3D objects in Figures 6C and 6D, with a regular RGB image shown for comparison in Figure 6A.

#### Going Beyond the Traditional Approach With Deep Learning

Advancements in deep learning are opening more possibilities to improve the IADA system by merging some steps (or even all steps) of the multistep pipeline into a single model, which can be fine-tuned as a whole process. Due to the rise in IADA research with the emergence of advanced algorithms, we can only highlight a few reports that demonstrate the gradual enhancements in IADA in this paper.

In 2015, Myers et al [27] from Google proposed the Im2Calories system, using deep learning for all stages of IADA. The classifiers are based on the GoogLeNet architecture, and the classification results are used to improve the semantic segmentation handled by the DeepLab network. For volume estimation, a new CNN architecture, trained with an RGBD dataset, estimates the depth map from a single RBG image and then converts the depth map to volume in the final step. Although the absolute error for some test foods could exceed 300 ml, the overall volume estimation results were deemed acceptable. The system still requires a food composition database to determine the nutritional values of the food in the final step.

The idea of using deep learning to estimate food volume is gaining popularity, and several systems are transitioning to using deep learning algorithms to estimate food volume without the need for an actual ToF sensor. In 2017, carbohydrate counting algorithms named Diabetes60 were proposed by Christ et al [28]. The system reported food-specific portions called “bread units,” which are defined to contain 12 to 15 grams of carbohydrates. This definition closely resembles the “carb unit” widely used in the diabetes field or the “exchange unit” in dietetic practice. The system was based on ResNet50 and trained using an RGBD image dataset that contained human-annotated bread unit information. It achieved a root mean square error of 1.53 (approximately 18.4-23 g of carbohydrate), while humans could achieve a root mean square error of 0.89 (approximately 10.7-13.4 g of carbohydrate) when compared with the ground truth. The modified ResNet was also used for fruit volume estimation, achieving an error of 2.04% to 14.3% for 5 types of fruit and 1 fruit model [94]. Furthermore, Jiang et al [95] introduced a system to classify liquid levels in bottles into 4 categories: 25%, 50%, 75%, and 100%. Using their own designed CNN architecture, they achieved a 92.4% classification accuracy when the system was trained with 3 methods of data augmentation. Furthermore, the system could achieve 100% classification accuracy when the bottle images had labels removed.

One challenge in converting a single 2D image into a 3D object is the difficulty in capturing the back side of an object in single-view images due to factors such as view angle or occlusion. Therefore, the food volume may be underestimated. Point2Volume was introduced in 2020 by Lo et al [96] to address the limitations. The system builds upon 2 of their previous works: a deep learning view synthesis [97] and a point completion network [98]. When a single-depth image is captured, a Mark region-based CNN—a combination of object detection and instance segmentation network—performs classification and segmentation, obtaining only partial point clouds due to occlusion. It then reconstructs the complete shapes and finally estimates the food volumes. This system demonstrated a volume estimation error of 7.7% for synthetic foods and 15.3% for real foods.

While the estimation of exact food volume has improved recently, dietitians and nutritionists often use a different approach. They compare unknown food amounts with known reference volumes, such as a thumb, matchbox, tennis ball, deck of cards, or a series of known portion-size images. Yang et al [99] introduced a system that mimics this mental estimation approach in 2021. The system classifies the unknown portion object to match the system’s set of reference volumes and then fine-tunes the predicted volume using the selected set. The system achieved a mean relative volumetric error of around 11.6% to 20.1% for their own real food image dataset. Interestingly, they noted that even when the system chose the wrong set of reference volumes—due to top-1 accuracy being <50% in most cases—the mean relative volumetric error still remained acceptable, implying that fewer reference volume sets might be sufficient.

Another crucial question is how many food classes should be included in the system to achieve usability in day-to-day situations. The goFood system [58], successor to the previous carbohydrate estimation system GoCARB, takes a different approach to expand the coverage beyond their included food classes. Using a modified Inception V3 architecture to classify food into a 3-level hierarchical structure: 18 types of generic food (eg, meat, bread, and dairy), 40 types of subgroups (eg, white bread and red meat), and 319 types of specific foods. This strategy mirrors the concept of a food exchange list, allowing the handling of a large number of foods without the need for an extensive number of fine-grained classifications. This lowers the number of unidentified food objects and results in achieving at least a 3% higher accuracy for food identification than the single-level Inception V3 classifier. Their newer 3D reconstruction algorithm, incorporating gravity data from the smartphone’s inertial measurement unit (eg, accelerometer or gyroscope), achieved a mean absolute relative error of 19%, surpassing the algorithm in GoCARB, which had 22.6% error.

Furthermore, CNN and deep learning could potentially estimate nutrients directly without relying on food composition tables, enabling an end-to-end approach for IADA. The originality of this method is unclear, but to the best of our knowledge, the first well-documented system was introduced by Miyazaki et al [100] in 2011. This system extracts 4 features from food images and estimates calories from these features instead of relying on food identification, portion estimation, and food composition tables as in multistage IADA. The system achieved a relative error of approximately 20% for 35% of items and 40% for 79% of items, which is relatively high. This idea inspired subsequent works by Ege and Yanai [29] from UEC in 2017. They applied a multitask CNN, a technique where a model is trained to perform multiple tasks simultaneously, using visual geometry group-16 for feature extraction and a calorie-annotated image dataset for training. The CNN system achieved an estimation error of 20% for 50% of items and 40% for 80% of items in their Japanese food image dataset. However, the system assumed that each food image contained only 1 food item; this limitation was addressed in their later works [101,102]. Multitask CNNs can be fine-tuned for the entire algorithm rather than for each stage as in a multistage architecture. This gives them the potential to surpass multistage architectures, similar to how deep learning and CNNs have outperformed handcrafted food identification algorithms. Therefore, they have gained significant attention from researchers [103-107].

Not only multitask CNNs but also generative adversarial networks, which are the backbone of image generation AI, such as Dall-E (OpenAI), can be used to learn the energy distribution map and estimate food energy directly from a single RGB image. Fang et al [30] from the TADA team applied this approach and achieved a mean energy estimation error of 209 kcal. Their subsequent work, which included adding food localization networks, improved accuracy by approximately 3.6% [108]. While most system predictions focus on food portions (volume or weight), calories, or macronutrients such as carbohydrates, in 2019, Situju et al [109] used a multitask CNN to predict the salt content of 14 types of food. This was achieved by training the multitask CNN with a dataset annotated for both calories and salt. The relative estimation error was 31.2% (89.6 kcal) for calories and 36.1% (0.74 g) for salt. These works provide evidence that advanced deep learning techniques yield promising results and offer room for improvement in IADA, garnering increasing attention from researchers today.

### Advancements and Challenges From the Dietitian’s Perspective

#### Overview

According to recently published information, both image classification and volume estimation techniques are comparable in accuracy to those of untrained humans or even trained professionals in some situations [92,110]. Some limitations exist, however, in relying on traditional methods, which indicates that another auxiliary system might be necessary to improve the overall accuracy and usefulness of a future developed system.

#### Using Recipe-Specific Nutritional Values

Currently, most existing systems rely on standard food composition tables to calculate the representative nutritional values of foods. While the United States Department of Agriculture National Nutrient Database is considered comprehensive, in practical dietetics, it is important to use recipe-specific nutritional values when available. For example, differentiating between a Subway sandwich (Subway IP LLC) and a Starbucks sandwich (Starbucks Corporation) using a food identification system may be feasible with a large image dataset of these specific sandwiches. However, it could be more straightforward to use location data to determine the brand of the sandwich.

Furthermore, when a food product has a nutrition facts label, it is essential to obtain the representative values directly from the label instead of relying solely on food composition tables. This can be accomplished either through a system equipped with optical character recognition or by accessing a vast nutrition facts label database, such as Open Food Facts [111]. By incorporating these recipe-specific and label-based nutritional values, the accuracy and relevance of food nutrient assessment systems can be significantly improved.

#### Challenges With Density Determination

The conversion of volume to weight in volume estimation approaches relies on food-specific density values, which can pose technical difficulties [112]. Furthermore, food-specific density is not provided in all food compositions; therefore, it must be obtained through calculation. Most food composition tables provide nutrient content per 100 grams of edible food, as it is derived from direct chemical analysis procedures. By contrast, food portion sizes are often measured in household units, such as teaspoons, tablespoons, or measuring cups.

The portion-specific weight must be divided by the standard volume of the household unit to calculate density. For example, according to the Thai food composition table, cooked mung bean sprouts weigh 78 and 34 grams for 1 serving (240 mL) and 1/3 serving (80 mL), respectively. This results in food-specific densities of 0.325 and 0.425 g/mL. However, relying on a single representative density value may not be appropriate, as it can contribute to overall system errors beyond just volume estimation. To address this challenge, a calibration curve-like method should be used instead of relying on a single density value. The accuracy and reliability of volume estimation systems can be improved, thus ensuring more precise and consistent results.

#### Guessing Missing Information

When assessing food intake, dietitians and nutritionists often encounter situations where certain food items are not readily available in food composition tables or nutrition databases. In such cases, a comprehensive analysis of the food needs to be conducted, breaking it down into its individual components. Using plain fried rice with egg as an example, the 2 cups of fried rice should be divided into at least 2 components: steamed white rice and chicken egg, which are visible in the image. However, additional components, such as seasonings and cooking oil, must be estimated. Seasonings, such as salt, soy sauce, and sugar, are typically added to enhance flavor, while cooking oil is often used to prevent food from sticking to the pan and to aid in the cooking process. Furthermore, the amount of seasoning and cooking oil may vary based on the personal experience or preference of the nutritionist who analyzes the food. Consequently, in nutrition research, it is recommended to have at least 2 or 3 analysts to reduce individual bias [113]. Using algorithms, which are based on standardized criteria, the variation caused by personal experience and subjectivity can be reduced.

#### Explainable System and Trust Issues

Using AI in health care has attracted close attention from health care communities worldwide, raising concerns about how to trust unexplained systems [114-116]. This concern is also shared by nutrition professionals. The black-box nature of deep learning algorithms makes it difficult for users to identify incorrect outputs.

When dietitians and nutritionists review a participant’s food photo and the estimated calorie intake is lower than expected, it could be due to underreporting or misreporting by the participant, selection of an inappropriate food item, forgetting to include certain amounts of oil in recipe analysis, or underestimating portion sizes. Dietitians and nutritionists can easily identify these errors. However, if the system only provides calorie outputs without additional information, it fails to establish trust with the users. Consequently, involving nutrition professionals in the development and evaluation of these systems is crucial to build trust and ensure that the technology meets their requirements.

## Discussion

### Principal Findings

In this study, we investigated the AI techniques used for IADA and analyzed the available literature to identify the principal findings in this field. Our scoping review encompassed 522 articles, and after careful evaluation, we included 84 (16.1%) articles for analysis, spanning from 2008 to 2021. After 2015, the increase in the number of published articles in this field can be attributed to various factors, including the growing availability of large datasets, advancements in AI development frameworks, and improved accessibility of hardware resources for AI-related tasks.

The principal findings were categorized into 2 main areas: food identification and food volume estimation. The chronological presentation of the articles allowed a better understanding of the algorithms’ complexity and the improvements achieved in accuracy. The transition from handcrafted food identification algorithms to deep learning-based algorithms occurred within a relatively short span of 5 years. This shift demonstrated the transformative power of deep learning in enhancing the accuracy and efficiency of food identification in image-based dietary assessment. Regarding food volume estimation, 4 different approaches were identified. However, all of these approaches share the common goal of translating 2D object views into 3D representations within a computer system and then converting these to weight to estimate representative nutritional values from a food composition table. While these approaches each have their strengths and limitations, the use of depth cameras is straightforward for measuring volume with fewer assumptions and might result in the lowest error rates compared with other methods. Nonetheless, the limited availability of depth cameras in some smartphones poses a significant challenge for implementing this approach. However, recent advancements in deep learning techniques offer promising alternatives to overcome the need for specific hardware to estimate volume and even directly estimate nutritional values without using a food composition table.

### Comparison With Prior Work

During our search for relevant studies, we encountered several review articles published before ours. Gemming et al [117] organized notable studies from the early stages of IADA development. Doulah et al [118] primarily focused on computational methods for determining energy intake, including IADA techniques and wearable devices aimed at replacing traditional dietary assessment methods. Lo et al [119] provided detailed explanations of techniques for both food recognition and volume estimation used in IADA studies. The survey from Subhi et al [120] and the systematic review from Dalakleidi et al [121] offer comprehensive comparisons of IADA systems, organized based on the subtasks of multistage architecture. Tay et al [122] provided an exclusive report on computational food volume estimation. While these review articles provide extensive information, they may be difficult to comprehend for nontechnical individuals, such as dietitians and nutritionists. This review is tailored to serve as a starting point for those who may not be familiar with the technical terminology and complexity associated with this field, presenting information in clear chronological order for easy following and comparison.

### Strengths and Limitations

While technology has advanced rapidly over the past 2 decades, it is important to acknowledge that some of the studies included in our review may have become outdated in terms of algorithm complexity, measurement techniques, and the accuracy of predicted results. Nonetheless, the findings from these earlier studies remain crucial from a dietitian’s perspective and provide valuable insights for future research and solution development. Although our search strategy was comprehensive and systematic, it is important to acknowledge that there may be studies that we were unable to identify or include in this study. Despite this limitation, our analysis provides a comprehensive overview of the principal findings in the field of IADA, shedding light on the potential and challenges of incorporating AI techniques into this domain.

### Conclusions

The application of AI has demonstrated promising results in enhancing the accuracy and efficiency of IADA. Advanced technologies, such as deep learning, CNNs, multitask CNNs, and generative adversarial networks, have significantly improved digitization of dietary intake. However, despite their potential, there are still challenges to overcome when implementing these technologies in real-world settings. To achieve broader coverage and increased reliability, integrating various inputs, such as food barcodes, direct label readers through optical character recognition, and location-specific recipes, could enhance the capabilities of IADA systems.

Additional research and development efforts are needed to address persistent issues, such as the limited availability of depth cameras, interassessor variation, missing information, and density estimation. While AI-based approaches offer valuable insights into dietary intake, it is essential to recognize that they were not designed to capture long-term usual intake entirely, which could be determined by aggregating self-reported and objective measures of dietary intake.

Furthermore, combining usual intake with additional aspects of health, such as physical activity, sleep patterns, and body composition, is required for a comprehensive understanding of the relationship between lifestyle, health, and disease. By overcoming these challenges, AI-based approaches have the potential to revolutionize dietary assessment and contribute to a better understanding of an individual’s intake, eating patterns, and overall nutritional health.

## Appendix

Multimedia Appendix 1 PRISMA-ScR (Preferred Reporting Items for Systematic Reviews and Meta-Analyses Extension for Scoping Reviews) checklist.

## Abbreviations

24HR: 24-hour dietary recall
AI: artificial intelligence
AR: augmented reality
CNN: convolutional neural network
EFR: estimated food record
IADA: image-assisted dietary assessment
PRISMA-ScR: Preferred Reporting Items for Systematic Reviews and Meta-Analyses Extension for Scoping Reviews
ResNet: residual network
RGB: red-green-blue (color model based on additive color primaries)
RGBD: red-green-blue with depth
SVM: support vector machine
TADA: Technology Assisted Dietary Assessment
ToF: time-of-flight
UEC: University of Electro-Communications

## Data Availability

All data generated or analyzed during this study are included in this published article.
